# Complete Genome Sequence of the Epidemic and Highly Virulent CTX-M-15-Producing *H*30-Rx Subclone of *Escherichia coli* ST131

**DOI:** 10.1128/genomeA.00988-13

**Published:** 2013-12-05

**Authors:** Paal S. Andersen, Marc Stegger, Maliha Aziz, Tania Contente-Cuomo, Henry S. Gibbons, Paul Keim, Evgeni V. Sokurenko, James R. Johnson, Lance B. Price

**Affiliations:** Microbiology and Infection Control, Statens Serum Institut, Copenhagen, Denmarka; Pathogen Genomics Division, Translational Genomics Research Institute, Flagstaff, Arizona, USAb; U.S. Army Edgewood Chemical Biological Center, Aberdeen, Maryland, USAc; Center for Microbial Genetics and Genomics, Northern Arizona University, Flagstaff, Arizona, USAd; Department of Microbiology, University of Washington School of Medicine, Seattle, Washington, USAe; Veterans Affairs Medical Center and University of Minnesota, Minneapolis, Minnesota, USAf; Department of Environmental and Occupational Health, George Washington University, Washington, DC, USAg

## Abstract

We report the complete genome sequence, including five complete plasmid sequences, of *Escherichia coli* ST131 isolate JJ1886. The isolate was obtained in 2007 in the United States from a patient with fatal urosepsis and belongs to the virulent, CTX-M-15-producing *H*30-Rx sublineage.

## GENOME ANNOUNCEMENT

*Escherichia coli* sequence type 131 (ST131) has emerged as one of the most prevalent extraintestinal pathogenic *E. coli* lineages in circulation today (1). The ST131 *H30* lineage dominates among fluoroquinolone-resistant and extended-spectrum β-lactamase (ESBL)-producing *E. coli* strains and is associated with recurrent urinary tract infections, pyelonephritis, and sepsis (1, 2, 3, 4, 5). Phylogenomic analysis indicated that the dominant ST131 ESBL gene, *bla*_CTX-M-15_, is associated with an *H*30 sublineage, designated *H*30-Rx (6). Here, we present the complete genome sequence of JJ1886, an ST131 *H*30-Rx isolate recovered from a patient with fatal urosepsis (7).

The genome sequence was assembled using 320 Mb of paired-end 100-bp HiSeq reads (Illumina, Hayward, CA), 225 Mb of 500-bp reads from the Roche Genome Sequencer FLX (Roche Diagnostics, Switzerland), and 187,000 reads (296 Mb) from the PacBio RS platform (Pacific Biosciences, Menlo Park, CA). The PacBio sequence reads were error corrected using HiSeq data (8). Sequences were assembled using multiple *de novo* assemblers, including MIRA (9), ABySS (10), and CLCbio (CLCbio, Denmark). Initial assembly of the Roche and HiSeq data yielded 52 chromosomal contigs after extension using PBJelly and PAGIT (11, 12). PacBio reads further reduced the number of chromosomal contigs to 18. Gaps were closed *in silico* using Genomics workbench 6.0.4 (CLCbio) by a combination of comparisons to *E. coli* reference genome sequences, with manual curation using HiSeq and PacBio data and verification using an optical map (OpGen, MD). Five complete plasmids were identified based on BLAST analysis and were verified using Illumina MiSeq sequencing (250-bp paired end) on isolated plasmids. The genome sequence was annotated using RAST (13).

The complete genome of JJ1886 comprises a 5,129,938-bp chromosome with a GC content of 50.8%, with 5,086 coding sequences, 88 tRNAs, and 22 rRNA features, plus five plasmids, pJJ1886-1 through pJJ1886-5, of sizes 1.6, 5.2, 5.6, 56, and 110 kb, respectively. According to ResFinder 1.4 (14), only pJJ1886-5 carries genes for resistance, including resistance to aminoglycosides and fluoroquinolones [*aac*(*6*′)*-Ib*, *aac*(*6*′)-*Ib*-*cr*, *aac*(*6*′)*-31* (~85% sequence homology)], beta-lactams (*bla*_OXA-30_, *bla*_TEM-1_), and chloramphenicols (*catB3*). However, the ESBL gene *bla*_CTX-M-15_ was integrated into the JJ1886 chromosome via an incomplete Tn*3* transposable element embedded within a lambda-like 58-kb prophage (identified by PHAST analysis [15]). This is in contrast with the only other complete, published ST131-*H*30 genome sequence, that of NA114 (isolated from a patient with prostatitis [16]), which likely has *bla*_CTX-M-15_ integrated into a previously described plasmid (17).

JJ1886 is the first complete genome sequence for a urosepsis ST131 isolate that includes all plasmids and has a chromosomal *bla*_CTX-M-15_ integration site. The genome sequence will serve as a valuable resource for studies on the epidemiology and pathogenicity of the highly virulent ST131 lineage.

### Nucleotide sequence accession numbers.

The complete sequences of the chromosome of *E. coli* JJ1886 and its five plasmids, pJJ1886-1 through pJJ1886-5, have been deposited in GenBank (accession numbers CP006784, CP006785, CP006786, CP006787, CP006788, and CP006789, respectively).
